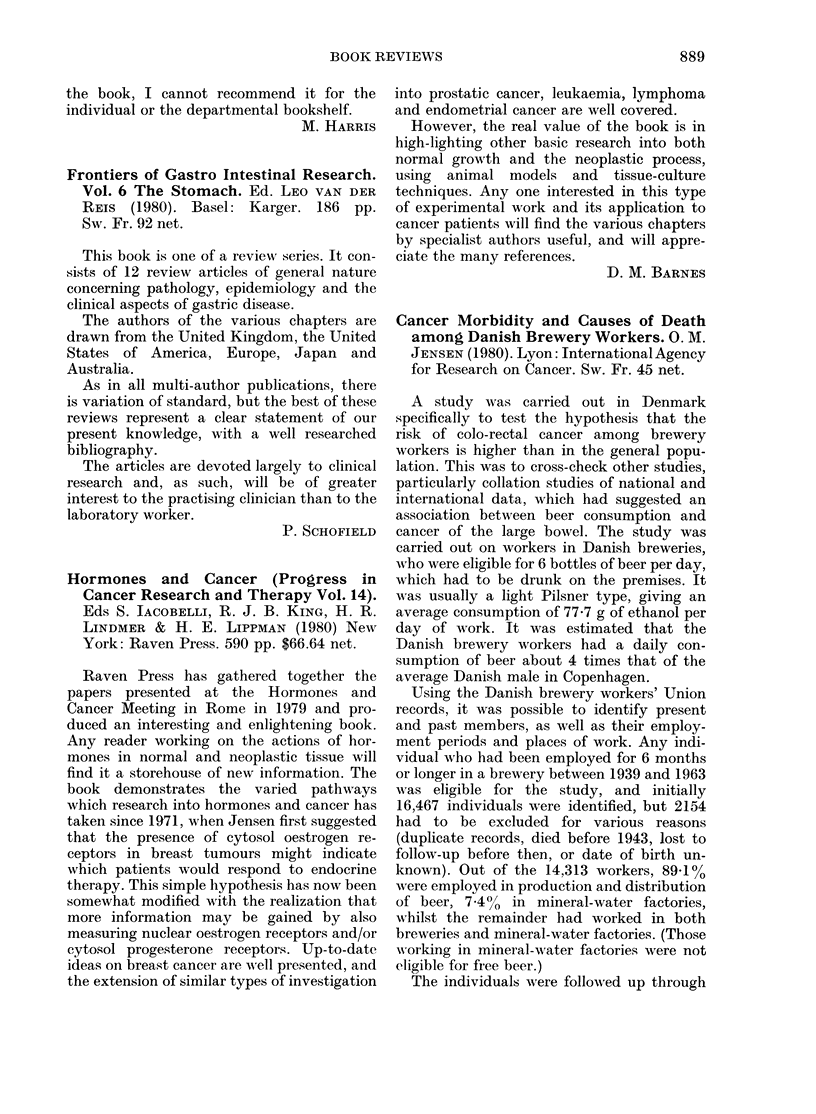# Frontiers of Gastro Intestinal Research. Vol. 6 The Stomach

**Published:** 1981-06

**Authors:** P. Schofield


**Frontiers of Gastro Intestinal Research. Vol. 6 The Stomach.** Ed. LEO VAN DER REIS (1980). Basel: Karger. 186 pp. Sw. Fr. 92 net.

This book is one of a review series. It consists of 12 review articles of general nature concerning pathology, epidemiology and the clinical aspects of gastric disease.

The authors of the various chapters are drawn from the United Kingdom, the United States of America, Europe, Japan and Australia.

As in all multi-author publications, there is variation of standard, but the best of these reviews represent a clear statement of our present knowledge, with a well researched bibliography.

The articles are devoted largely to clinical research and, as such, will be of greater interest to the practising clinician than to the laboratory worker.

P. SCHOFIELD